# A minimally invasive transfer method of mesenchymal stem cells to the intact periodontal ligament of rat teeth: a preliminary study

**DOI:** 10.3906/biy-1712-62

**Published:** 2018-10-25

**Authors:** Nisa GÜL AMUK, Gökmen KURT, Melis KARTAL YANDIM, Aysun ADAN, Yusuf BARAN

**Affiliations:** 1 Department of Medical Biology, Faculty of Medicine, İzmir University of Economics , İzmir , Turkey; 2 Department of Molecular Biology and Genetics, Faculty of Life and Natural Sciences, Abdullah Gül University , Kayseri , Turkey; 3 Department of Molecular Biology and Genetics, Faculty of Science, İzmir Institute of Technology , Urla, İzmir , Turkey; 4 Department of Orthodontics, Faculty of Dentistry, Bezmiâlem Vakıf University , İstanbul , Turkey; 5 Department of Orthodontics, Faculty of Dentistry, Erciyes University , Kayseri , Turkey

**Keywords:** Stem cell, periodontal ligament, transfer without scaffold, green fluorescent protein, cyclooxygenase-2

## Abstract

The aim of this study was to introduce a minimally invasive procedure for mesenchymal stem cell (MSC) transfer into the intact periodontal ligament (PDL) of the molar teeth in rats. Ten 12-week-old Wistar albino rats were used for this preliminary study. MSCs were obtained from bones of two animals and were labeled with green fluorescent protein (GFP). Four animals were randomly selected for MSC injection, while 4 animals served as a control group. Samples were prepared for histological analysis, Cox-2 mRNA expression polymerase chain reaction analysis, and fluorescent microscopy evaluation. The number of total cells, number of osteoclastic cells, and Cox-2 mRNA expression levels of the periodontal tissue of teeth were calculated. The number of total cells was increased with MSC injections in PDL significantly (P < 0.001). The number of osteoclastic cells and Cox-2 mRNA expression were found to be similar for the two groups. GFP-labeled MSCs were observed with an expected luminescence on the smear samples of the PDL with transferred MSCs. The results of this preliminary study demonstrate successful evidence of transferring MSCs to intact PDL in a nonsurgical way and offer a minimally invasive procedure for transfer of MSCs to periodontal tissues.

## 1. Introduction

Periodontal ligament (PDL) has many functions such as supporting and providing nutrition to teeth, homeostasis, and repairing damaged tissues. Resorption or loss of cementum, degeneration of alveolar bone and/or gingiva due to destructive periodontal diseases, or root resorption after trauma are regenerated by heterogeneous cell populations of PDL (Murakami et al., 2003) . These cells are capable of differentiating into cementoblasts, osteoblasts, or other connective tissue cells (Isaka et al., 2001) . The repair capacity of PDL through these cells indicates that PDL contains progenitor cells (Beertsen et al., 1997) that wait in the tissue and act as needed. Recent investigations have shown that these progenitor cells consist of stem cells (Behnia et al., 2012) and, PDL stem cells has been found to have high similarity with mesenchymal stem cells (MSCs) (Kramer et al., 2004) .

For the treatment of periodontal defects, stimulation of the reparative effects of stem cells in PDL by transferring additional MSCs to the defect region has been achieved, and the regenerative effects of MSCs transfer have been reported in previous animal studies (Kawaguchi et al., 2004; Hasegawa et al., 2006; Liu et al., 2008; Tsumanuma et al., 2011; Takewaki et al., 2017) . However, these MSC applications were carried out by transfer of the cells into experimentally created periodontal bone defects with the purpose of imitation of the original defect structure and simulation of a surgical clinical approach, which aims to access the root surface for cleaning the affected root surface and removing granulation tissues. Hereby, the presence of a bone cavity near periodontal tissues such as in furcation defects or intrabony periodontal defects made possible the transfer of cells to the PDL. However, all dental pathologies may not have a defect cavity near the damaged periodontal area, like root resorption or dehiscence, or fenestration defects located on the thin vestibular alveolar bone. In these cases, stem cell application and delivery of cells to the thin, intact PDL tissue without using bone cavities is a challenging issue and such a MSC transfer approach has not been identified in the literature. For this purpose, infiltrative and intraligamentary anesthesia injection procedures may be used for effective material transfer. The material can reach the cervical, middle, and apical regions of the PDL and also can be released to the nearby apical tissues from the submucosal area through these injections. Although these injections are known as minimally invasive and painless dental injection methods (Galili et al., 1984; Achar and Kundu, 2002) , it might be considered that MSC transfer by injections may cause an inflammatory reaction in the related region. Uncontrolled inflammation is held accountable for periodontal diseases in which destruction progresses involving all periodontal tissues and causes loss of the supporting connective tissue, alveolar bone, and roots of teeth (Pihlstrom et al., 2005) . Therefore, transfer of therapeutic cells for regeneration of related tissues should not cause additional inflammation that can cause destruction.

Cox-2 is an important marker of inflammation (Seibert et al., 1994) , and identifying Cox-2 expression levels demonstrates the presence of inflammation in the related region. Multinuclear cell activation is secondary to the Cox2 expression. Multinuclear cells have specific isoenzymes in their cytoplasm that resist inhibition by tartaric acid. Tartrate-resistant acid phosphatase (TRAP) is expressed by osteoclasts, macrophages, dendritic cells, and a number of other cell types (Hayman, 2008) . Using this mechanism, TRAP staining has been developed as an advanced method for determining multinuclear cells (Liu et al., 2011) . Detection of the presence or count of multinuclear cells and expression of Cox-2 may provide information as to whether inflammation exists in the PDL or not.

MSCs have the ability to take part in many biological events in the PDL. If transfer of stem cells to the intact PDL tissue is achieved without inflammatory and destructive effects in a sufficient amount, the repair or regeneration of the targeted alveolar bone, cement, and connective tissue can be more successful in gene therapy and tissue engineering applications. Various transfer methods of stem cells to the periodontal tissues using bony cavities existing in intrabony or furcation periodontal diseases have been reported, but an easy, minimally invasive method that does not involve minor or major surgical interventions like incision, flap raising, or osteotomy for delivering cells into the intact PDL with the purpose of treating pathologies like dehiscence, fenestration defects, or root resorption, which may occur especially during orthodontic tooth movement without defective cavities, has not been presented in the literature. Therefore, the aim of this study was to introduce a new and direct MSC transfer procedure to the intact PDL of rat molar teeth.

## 2. Materials and methods

### 2.1. Animals

Ten 12-week-old male Wistar albino rats with a mean weight of 268.26 ± 6.14 g were used in this preliminary study. Animal selection, management, and experiment protocol were approved by the Erciyes University Regional Animal Research Ethics Committee (Approval Code: 11/136). Four animals received only saline solution injections (control group), whereas four rats received MSC transfer injections (experimental group). Two rats were used for MSC isolation.

### 2.2. Isolation and culture of mesenchymal stem cells

**Figure 1:**
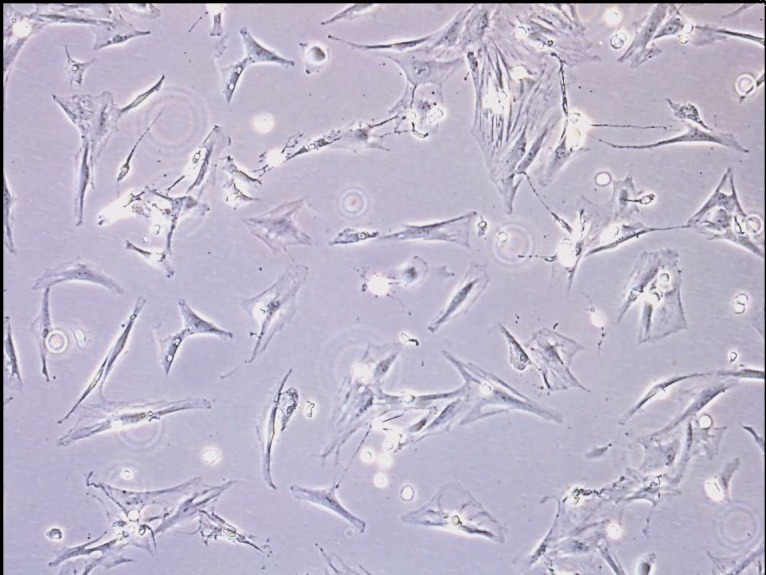
Microscope images of the cultured bone marrow-derived mesenchymal stem cells before transfection (magnification: 100×).

The femurs and tibias of rats were dissected away from attached muscle and connective tissue. Dulbecco’s modified Eagle medium (DMEM; Biological Industries, Israel) was passed through the bones using an 18-gauge needle. The marrow was collected in a Falcon tube and then centrifuged at 1000 rpm for 10 min. The pellet was suspended and cultured in DMEM containing 15% fetal bovine serum (FBS) and 1% penicillin-streptomycin (37 °C and 5% CO2 incubator). The cultured bone marrow MSCs were observed under a microscope in order to monitor their expansion and morphology (Figure 1). When 80% to 90% confluence was obtained, cells were trypsinized using Trypsin/EDTA solution and cells were seeded onto fresh plates.

### 2.3. Characterization of bone marrow MSCs

**Figure 2:**
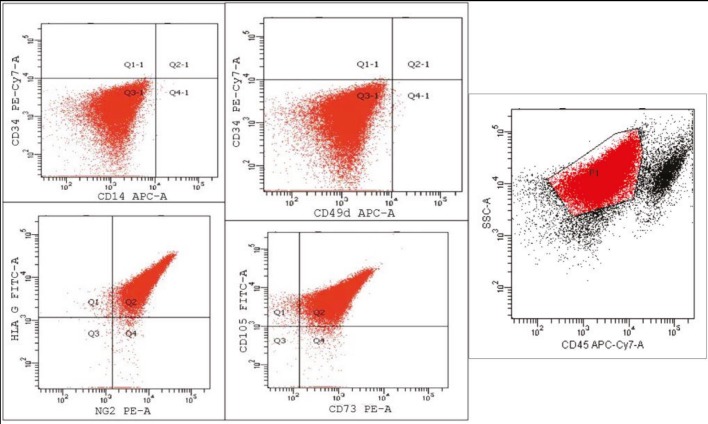
Characterization of the mesenchymal stem cell population from rat bone marrow. Representative flow cytometry dot plots showing the principal mesenchymal stem cell markers. The significant expressions of CD105, CD73, and NG2 that are specific to MSC antigens, and no detection of CD45, which is specific to the hematopoietic marker antigen, indicated that these cells are MSCs.

MSCs were characterized at the second passage. The cells were incubated with antibodies against CD45 PC5, CD73 PE, CD105 FITC, and NG2 PE. A total of 20,000 cells/ sample at a flow rate of approximately 200 cell events/s were recorded to obtain fluorescence histograms. A Coulter Epics XL-MCL was used during the experiments and the data were analyzed using EXPO 32 ADC software (Beckman Coulter Inc., USA). Flow cytometry analysis revealed that there were significant expressions of CD105, CD73, and NG2, which are specific to MSC antigens, while there was no detection of CD45, which is specific to the hematopoietic marker antigen (Figure 2). These results showed that these cells are MSCs.

### 2.4. Transfection of mesenchymal stem cells using green fluorescent protein (GFP)

**Figure 3:**
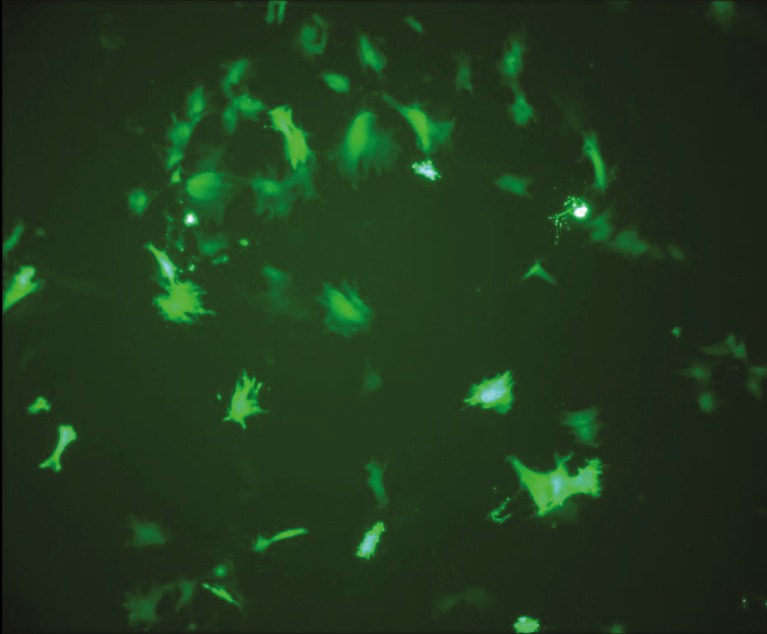
Green fluorescent protein (GFP) shining in fluorescent microscope images of mesenchymal stem cells transfected with GFP prior to injection (magnification: 40×).

In order to visualize the stem cells in vivo, cells were transfected with a pEGFP-N2 vector including a GFP encoding gene (ClonTech, USA) using the Metafectene Pro Transfection Reagent (Biontex Laboratories GmbH, Germany) at the third passage and 5 × 105 cells were grown at 37 °C and 5% CO2. On the day of transfection, blank DMEM was transferred onto the cells in the flasks. Plasmid DNA and the transfection reagent were mixed to form transfection complexes, and these complexes were transferred dropwise onto the cells. After 48–72 h, the transfected cells were visualized under a fluorescent microscope (Figure 3).

### 2.5. Transfer of MSCs to the rats

In order to transfer the MSCs to the PDL of the upper first molar of the experimental group of rats, injections were performed under general anesthesia on the first, sixth, and eleventh days of the experiment. On the same days, control group animals received saline solution injections. A specially designed intraoral retractor was produced in order to obtain adequate retraction of soft tissues. Thirty unit-insulin syringes with 29 gauge needle width and 8 mm needle length, which provided easy reach, were selected for the injections. The ends of the needle were inclined at 30° to facilitate access to the related intraoral region.

**Figure 4:**
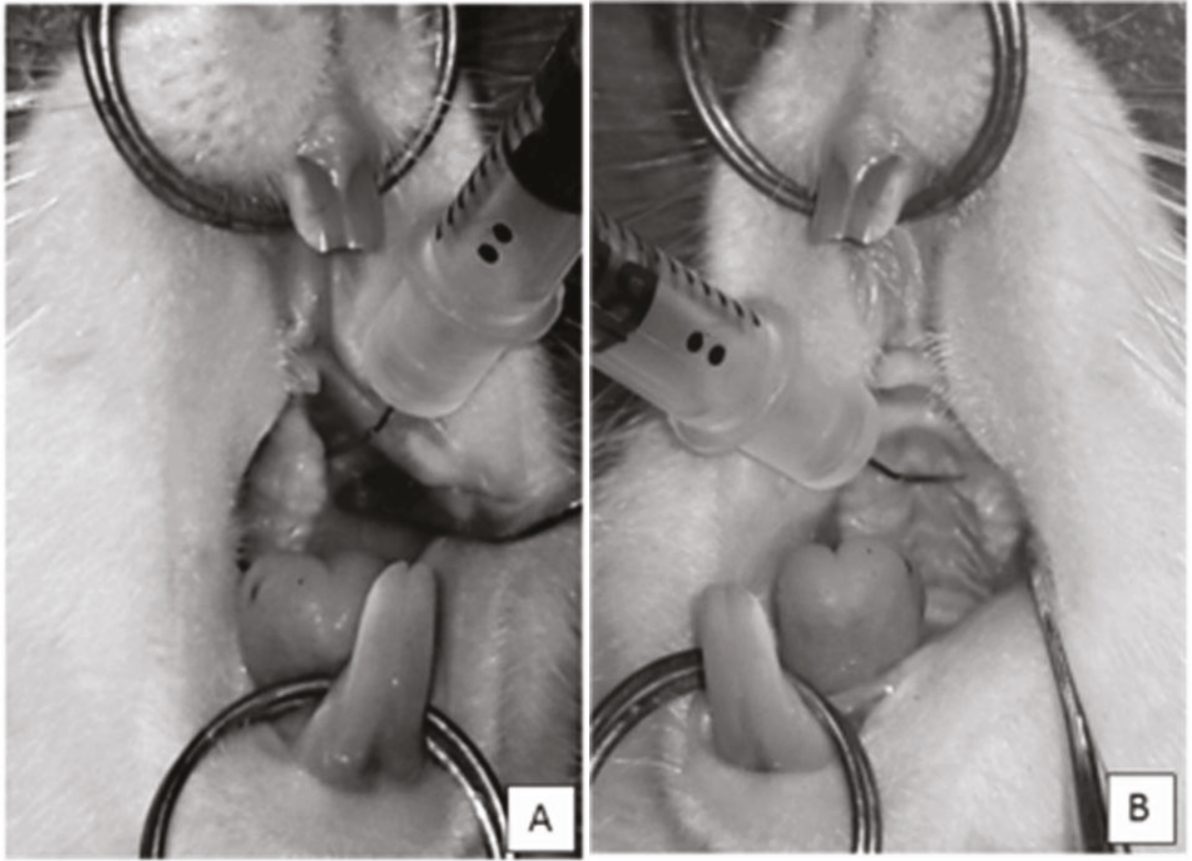
Injection of stem cells to the mesial of the upper first molars from the (A) mesiovestibuler and (B) mesiopalatal sides of teeth using 30 unit-insulin syringes with 29 gauge needle width and 8 mm needle length, inclined at 30° to facilitate access to the related intraoral region

For each tooth of the experiment group rats, 1.25 × 105 + 1.25 × 105 = 2.5 × 105 cells were homogenized in 0.025 mL of saline solution. Each injection to the mesial of the upper first molars was performed as infiltrative and intraligamentary anesthesia injections from the mesiovestibuler (1.25 × 105 cells) (Figure 4A) and mesiopalatal (1.25 × 105 cells) (Figure 4B) sides.

The homogeneity of the cells in transfer solution was ensured by using a vortex before every injection. Attention was paid not to create trauma in the application. The solution was injected smoothly and unpressurized. All injections were performed successfully and no bleeding or inflammation signs were observed after injections.

At the end of the thirteenth experimental day, the study and control group rats were euthanized with an overdose of anesthetics (200 mg/kg sodium-pentobarbital, Abbot, USA).

### 2.6. Fluorescent microscopy examination

In order to investigate the presence of the transferred GFP-tagged MSCs, samples were prepared for fluorescent microscopy examination.

**Figure 5:**
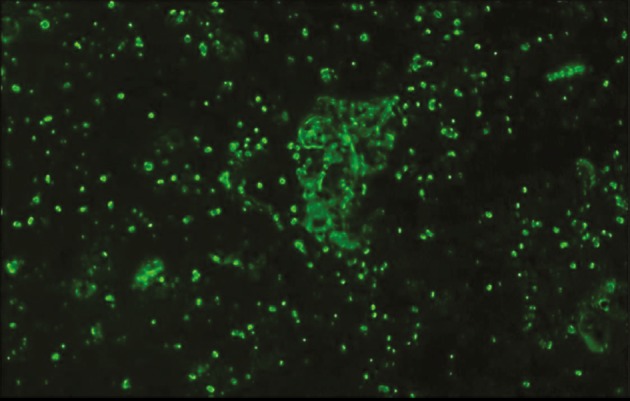
GFP shining on the fluorescent microscopy images of periodontal ligament smear sample of cell transfer group teeth after injection, indicating the presence of GFP-positive cells in the periodontal ligament of related teeth (magnification: 10×).

Upper first molars were extracted from the alveolar socket carefully. All roots of the extracted teeth were rubbed over the object plate entirely in order to obtain the PDL tissue smear. Periodontal tissue samples were transferred to the fluorescent microscopy unit in a dark closed box immediately. Samples were examined by fluorescent microscopy (Olympus BX52 Research System, Olympus Corp., Japan) in a dark room in order to detect GFP-positive cells in the PDL tissue of the upper first molars (Figure 5).

### 2.7. Histologic preparation and evaluation

The right and left posterior maxilla of two rats from each group were dissected and transferred to the histology laboratory in 10% formaldehyde. Segments were kept in ifxative for 24 h at 4 °C, rinsed, and decalcified at 4 °C for approximately 8 weeks.

**Figure 6:**
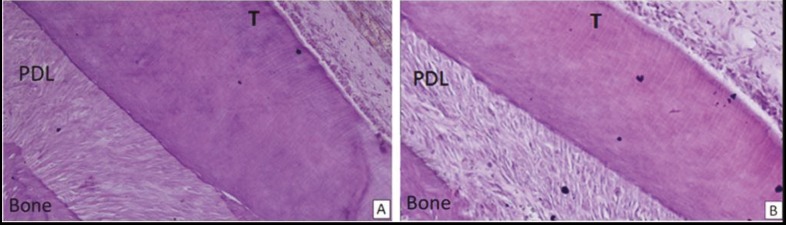
Light microscopic findings of hematoxylin and eosin staining of mesiobuccal root of the upper first molar teeth on the sagittal plane in (A) control tooth and (B) mesenchymal stem cell group showed the increased cellular density of the periodontal ligament in the cell-transferred teeth relative to control group teeth. Bone: Alveolar bone, T: tooth structures (dentin, cementum, pulp), PDL: periodontal ligament (magnification: 100×).

**Figure 7:**
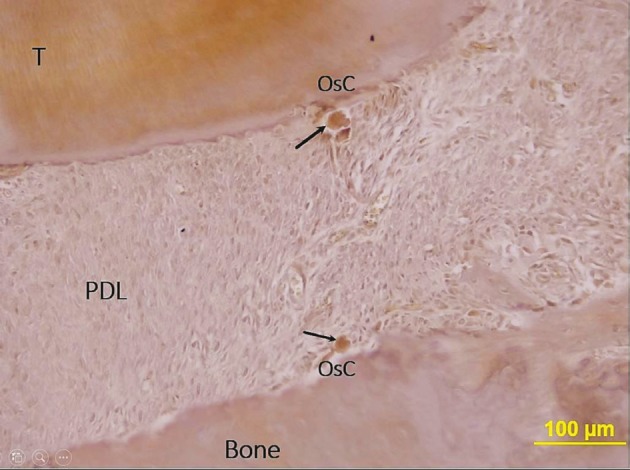
Tartrate-resistant acid phosphatase (TRAP)-staining of mesiobuccal root of the upper first molar teeth on sagittal plane. Black arrows indicate TRAP-positive cells (dark brown color) in the periodontal ligament (pale brown color) of control group teeth. Bone: Alveolar bone, T: tooth structures (dentin, cementum), PDL: periodontal ligament, OsC: osteoclastic cells.

The specimens were embedded in parafin and 5 µm parasagittal sections were cut and stained with hematoxylin-eosin (H&E) and TRAP. TRAP staining was performed using an Acid Phosphatase, Leukocyte (TRAP) Kit (Sigma-Aldrich Chemie GmbH, Germany). Staining was carried out according to manufacturer’s instructions. Diaminobenzidine (DAB, Sigma-Aldrich) was used for the color reaction during the TRAP staining, resulting in a brown color. Counterstaining was performed using Gill hematoxylin. Five adjacent H&E slides showing the longest length of the mesiobuccal root of the first molar were evaluated in terms of total cell count (Figures 6A and 6B), and the multinuclear cell count of the PDL was calculated on five adjacent TRAP staining slides (Figure 7). Photomicrographs were taken digitally with a microscope and digital camera system (Olympus CX41/ DP25; Olympus Corp.). Then photomicrographs of serial sections (magnification: 100×) were divided into grids (grid size: 200) using Adobe Photoshop CS3 version 10.0 for total cell count evaluation. The total cell count in each of the grids of the apical, middle, and cervical periodontal regions was calculated. The mean total cell count value of 5 adjacent slides was identified for each specimen.

The PDL area was divided into pieces on TRAP staining slides in order to calculate the number of TRAPpositive multinuclear cells existing in the PDL. The values for 5 sections were averaged for each tooth.

### 2.8. Determining expression levels of Cox-2

The upper right and left first molars were extracted from the alveolar socket carefully. The extracted teeth containing PDL tissue in their roots were placed in mRNA fixation solution. RNA was isolated from samples using a QIAamp RNA Blood Mini Kit (QIAGEN 52304). Samples were homogenized in 300 µL of buffer RTL and β-mercaptoethanol mix for 3 min. RNA isolates were dissolved in 30 µL of RNase-free water. cDNA was synthesized from RNA isolates with a First Strand cDNA Synthesis Kit (Thermo Scientific K1612). The obtained material was analyzed by real-time polymerase chain reaction (PCR) for Cox-2 mRNA expression assessment using the ACTB housekeeping gene for the internal control (Table 1). Ct values and the following formula were used for the data analysis: 2.012^[(Norm. Ct) – (GOI Ct)] (Norm. Ct: normalizer Ct value, with the ACTB gene (housekeeping gene) used as the normalizer; GOI Ct: gene of interest Ct value). The protocols for PCR (Rotor-Gene Q 6plex (QIAGEN, USA)) are presented in Table 2.

**Table 1:** Primers and probes used in real-time polymerase chain reaction.

Genes	Primers and probes	Sequences	Size (bp)	5’ modification	3’ modification
Rat_ACTB	Forward	AAGATGACCCAGATCATGTTTGAGACC	27	-	-
	Reverse	ATGCCACAGGATTCCATACCCAGG	24	-	-
	Probe	TCACCACCACAGCTGAGAGGGAAATCGT	28	FAM	BHQ-1
Rat_Cox-2	Forward	CCATGGGTGTGAAAGGAAATAAGGAAC	27	-	-
	Reverse	CACCGATGACCTGATATTTCAATTTTCC	28	-	-
	Probe	CATGATTTAAGTCCACTCCATGGCCCAGTC	30	ROX	BHQ-2

**Table 2:** Polymerase chain reaction components and amounts (μL).

PCR components	Amount
dH2O	16.8 μL
10X Buffer (Complete, Bioron GmbH)	2.5 μL
dNTP mix (each of 10 mM)	0.5 μL
DNA polymerase (SuperHot Taq, Bioron GmbH )	0.2 μL
Forward primer (5 μM)	1 μL
Reverse primer (5 μM)	1 μL
Probe (5 μM)	1 μL
cDNA	2 μL
Total	25 μL

### 2.9. Statistical analysis

All statistical analysis was performed using the SPSS 20.0 for Windows (IBM Corp., Armonk, NY, USA). The Mann–Whitney U test was used for comparison of the total cell count, multinuclear cell count, and Cox-2 mRNA expression levels of the groups. When the P-value was less than 0.05, the statistical test was determined as significant.

### 2.10. Post hoc power calculation

A post hoc power calculation (α = 0.001) was performed based on the number of total cell results. The power of the study was found greater than 95%.

## 3. Results

**Figure 8:**
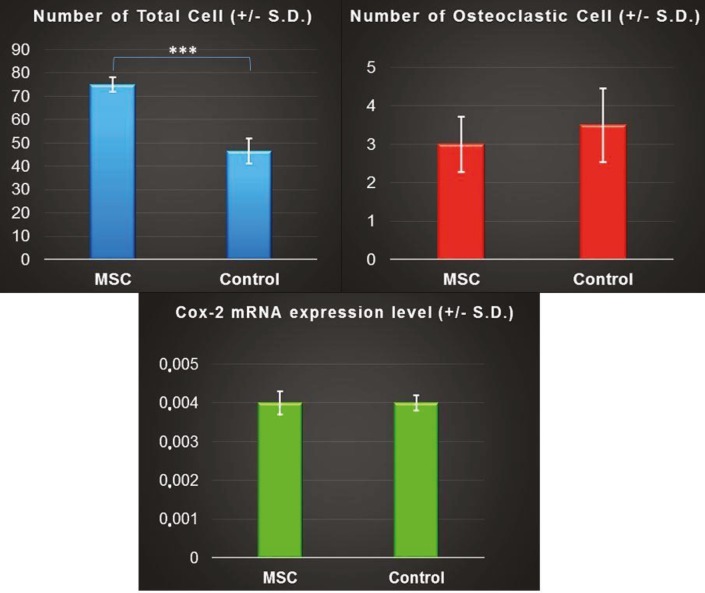
Bar graph representation of number of the total cells, number of osteoclastic cells, and Cox-2 mRNA expression level in mesenchymal stem cell (MSC) and control groups. *** P < 0.001.

H&E staining evaluation revealed that MSC application increased the cell count in the upper first molar PDL of MSC group rats. The number of total cells was calculated as 75 ± 3.02 cells/sample in the MSC group and 46.50 ± 5.32 cells/sample in the control group. The difference between the total cell values of the two groups was found to be statistically significant (P < 0.001) (Table 3; Figures 6A, 6B, and 8).

**Table 3:** Comparison of the number of total cells, number of osteoclastic cells, and Cox-2 mRNA expression levels between mesenchymal stem cell and control groups.

Groups	MSC			Control			P
	Median	25%	75%	Median	25%	75%	
NTC	75.00	72.50	76.00	46.50	41.00	48.50	***
NOsC	3.00	2.00	3.50	3.50	2.00	4.00	NS
Cox-2	0.004	0.002	0.006	0.004	0.003	0.007	NS

Nonnormally distributed data presentation with the first quartile (25%), second quartile (median), and third quartile (75%). MSCs: Mesenchymal stem cells, NTC: number of total cells, NOsC: number of osteoclastic cells, Cox-2: cyclooxygenase-2 mRNA expression level, NS: nonsignificant, ***: statistically significant difference at P < 0.001.

The other observations on H&E staining slides were related to the structural integrity assessment of the PDL and distribution of transferred cells through the ligament. The structural integrity of the alveolar bone, PDL, and root surface was maintained for both groups. No destruction area or tissue loss was observed in the MSC and control group samples. Histomorphometric evaluation was carried out at three vertical levels, as in the grids of the apical, middle, and cervical periodontal regions. All three grids contained similar numbers of cells for both groups. This method of cell calculation provided an idea about the distribution of cells in the PDL, especially for MSC group samples. The similar cell count in the PDL at the apical, middle, and cervical periodontal regions revealed that transferred MSCs were probably distributed homogeneously throughout the PDL.

In the TRAP staining evaluation, the number of osteoclastic cells in the MSC group (3 ± 0.72 cells/sample) and control group (3.50 ± 0.96 cells/sample) did not exhibit statistically significant differences between each other (Table 3; Figure 8). Transferred MSCs showed marked luminescence in the fluorescent microscopy examination of PDL samples (Figure 5) and thus the presence of GFPlabeled MSCs in the PDL was proven. PCR analysis results revealed that the Cox-2 expression levels of the MSC group (0.004 ± 0.0003) and control group (0.004 ± 0.0002) did not show any statistically significant difference (Table 3; Figure 8). The similar Cox-2 expression levels of the two groups may indicate no pain formation and/or no inflammatory response to MSC transfer by injections. In addition, other signs of inflammation were not observed in the injection areas, such as redness or tumors.

## 4. Discussion

The therapeutic effects of stem cell applications have been presented in medical sciences and in dentistry (Cartier et al., 2009) . The natural healing process works through various mechanisms and stem cell reserves of tissues play an important role in this system. Additional stem cell applications to the damaged tissues activate cellular mechanisms and accelerate the healing process (Falanga et al., 2007) . PDL has great importance in production of new connective tissue attachments, new bone, and cement tissues by means of its regenerative potential (Beertsen et al., 1997; Isaka et al., 2001; Murakami et al., 2003; Behnia et al., 2012; Bright et al., 2015) .

The effects of stem cells on periodontal diseases have been investigated on experimental periodontal bone defects (Galili et al., 1984; Kawaguchi et al., 2004; Liu et al., 2008). The delivery of stem cells to the damaged areas of the periodontal tissues has been achieved by transfer of cells into cavities created by removing the related alveolar bone and other periodontal tissues surgically for imitation of clinical defect structures and scaffolds have been used as cell carriers. Yang et al. (2010) recorded improvement during the healing of rat periodontal defects after stem cell application with the transfer of 1 × 107 cells via microcarrier gelatin beads and reported regeneration of the bone, cementum, and PDL. Takewaki et al. (2017) experimentally generated a class III furcation defect on premolars of beagle dogs. MSCs in their own extracellular matrix were transplanted into bone defects with no artificial scaffold and periodontal tissue regeneration was reported. Baba et al. (2016) investigated the safety and efficacy of surgical implantation of autologous MSCs with a biodegradable three-dimensional woven-fabric composite scaffold and platelet-rich plasma on patients. As a result of this clinical study, the authors suggested that this procedure may be a safe and effective regenerative treatment option for periodontitis. These reports of preclinical and clinical studies present successful evidence for the regeneration of intrabony or furcation defects through stem cell applications and may support the clinical use of MSCs in such diseases. However, in the management of cases with root resorption, dehiscence, or fenestration defects there are not any existing cavities for transfer of MSCs with surgically approaches using carriers. Furthermore, although some periodontal defects including microbial destruction on the root surface and/or inflammatory granulation tissues around the teeth require cleaning of the affected root surface and removal of granulation tissues before regenerative periodontal therapies using MSCs, orthodontically induced inflammatory root resorption and/or vestibular alveolar bone loss such as dehiscence or fenestration defects did not occur because of any microbial attack or infection. Therefore, in such cases, the regeneration of damaged cement, bone, or PDL may not require surgical intervention because there is no need to clean any infected area and the inflammation is the result of orthodontic loading. Instead of invasive or surgical solutions, dental injections, which have been used for anesthetic purposes since the discovery of lidocaine (xylocaine) in 1943 (Achar and Kundu, 2002) , may be used as a beneficial method for cell transfer. The choice of minimally invasive nonsurgical methods would provide elimination of discomfort and postoperative pain associated with surgery as well as an increase of clinical efficiency and reduction in treatment cost.

Although different injection methods have been used for cell transfer with successful results to the expanded median palatal suture after rapid maxillary expansion in rats (Ekizer et al., 2015) , to surgically made cleft palate bone defects in rats (Tavakolinejad et al., 2014) , to the intraarticular area of chronic osteochondral defects in rabbits (Harada et al., 2015) , and to the intraarticular area of the temporomandibular joints in rats (Oyonarte et al., 2013) , direct cell transfer into the thin, intact periodontal ligament without presence of bone cavity or surgical preparation for the regeneration of damaged periodontal tissues has not been reported before to the best of our knowledge. Therefore, successful cell transfer was aimed to be carried out via intraligamentary and infiltrative dental injections targeting the PDL tissue. To achieve successful results, the number of cells that will be necessary for optimum activation in the targeted area should be estimated.

The dimension of the PDL area of the first molars in rats was estimated to be approximately 1.5 mm in width, 1.5 mm in depth, and 1.5 mm in length according to measurements of the area around the root area, so 2.5 × 105 cells were considered sufficient for this region. No carrier was used for delivery of cells through the intact PDL for this study, in order not to increase the volume of injected material. Cells were diluted in 0.025 mL of saline solution and cell transfer was performed via dental injections.

Nevertheless, some solution containing the cell material can be lost because of intraligamentary pressure, so more MSC solution was prepared than necessary.

Following these injections, the presence of cells in the PDL and related tissues was observed with histological and fluorescent microscopy images. GFP provides cell visualization in target tissues using small molecules that have fluorescent or luminescent properties. Thus, it has been used for studies of gene expression (Rizzuto et al., 1995) and offers excellent visualization of gene expression and protein localization in the related tissue.

A statistically significant increase in total cell count in the periodontal tissues of the experimental group was also accepted as evidence of successful cell transfer through injections. However, an increase in the cell number in the PDL can be attributed to both inflammatory or resorptive cells that migrated to this area after injection and/or MSCs that were transferred to the PDL by injection. Although possible tissue damage during injections can be avoided by feeling pressure back as the injection is made, using the needle carefully and injecting material smoothly into the PDL (Galili et al., 1984 ), two examinations were performed in order to detect the existence of inflammation in PDL tissue after MSC transfer by injection. The expression level of Cox-2 is a determinative marker of inflammation (Seibert et al., 1994) , so the mRNA expression level of Cox-2 was measured and compared between the two study groups. PCR results showed that expression levels of Cox2 in the MSC and control groups were not significantly different, indicating no inflammatory response indicated by Cox-2 after cell transfer.

Most studies reported the successful identification of multinuclear bone cells with TRAP staining (Tsumanuma et al., 2011) . In this study, multinuclear cells in the PDL could be observed clearly and the count of multinuclear cells was similar in the two groups. No significant differentiation or migration of multinuclear inflammatory cells was found after MSC injection. Furthermore, the antiinflammatory effects of stem cells have been shown in recent studies (Iyer and Rojas, 2008) , and these effects are an advantage of the MSC injection procedure that could provide more reparative cells and fewer multinuclear cells in the targeted area. After TRAP and PCR examination results, it can be stated that cell increase in the PDL is not a result of inflammation in the PDL region. These cells are mostly transferred MSCs, and GFP reflection of the PDL samples from the experimental group supports the existence of MSCs in the PDL. The high number of GFPpositive cells seen in smear specimens was also attributed to the collective nature of smear samples. The PDL around all roots of the same tooth was collected for obtaining each of the smear specimens.

Some cases of periodontal tissue destruction are closely related to orthodontic treatment (Vanarsdall, 1994) . Dental arch expansion and incisor buccal-lingual movement are accepted as the most serious orthodontic movements in terms of periodontal tissue loss (Vanarsdall, 1994) . Garib et al. (2006) reported that orthodontic palatal expanders reduced the buccal bone thickness of the maxillary posterior teeth and cause bone dehiscence of the anchorage teeth buccal aspect. Also, external apical  root  resorption  remains a  common  iatrogenic destruction in orthodontic practice. Based on the amount of orthodontic forces, the duration of treatment, the initial morphology of the alveolar bone, and the structure of the root and periodontal ligament, orthodontic treatments are accepted as predisposing to root resorption, gingival recession, crestal resorption, and alveolar defects (Vanarsdall, 1994) . If such problems are encountered during or after orthodontic treatment, conventional periodontal treatment approaches may result in inadequate periodontal tissue healing. Therefore, recent investigations have focused on advanced cellular therapy options because of the regenerative capacity and availability of stem cells (Kawaguchi et al., 2004; Hasegawa et al., 2006; Liu et al., 2008, Takewaki et al., 2017) . Accurate and efficient transfer of stem cells is one the most important steps for successful treatment results. In this regard, a minimally invasive and nonsurgical procedure for stem cell transfer into the intact PDL may be accepted as a promising transfer method for the treatment of root resorption, dehiscence, fenestration, or alveolar crestal defects where intact PDL should not be damaged by invasive or surgical stem cell transplantation attempts. Further investigations should be designed to evaluate the therapeutic effects of MSCs transferred to the PDL using the method defined above in orthodontically induced root resorption and alveolar bone defects like dehiscence, fenestration, and crestal resorption. Before clinical applications, advanced studies are needed to clarify the time-dependent local distribution pattern of transferred stem cells and how long the injected MSCs will exist in the intact PDL as an active reparative cell group.

In conclusion, the findings of this study show that MSCs transferred to the PDL of rat teeth through dental injection successfully reached periodontal tissues without an inflammatory response. The stem cell transfer method defined by this study is an important step toward achieving minimally invasive and nonsurgical therapeutic stem cell applications to the intact PDL of teeth.
